# A Curriculum-Based Approach to Teaching Biosafety Through eLearning

**DOI:** 10.3389/fbioe.2018.00042

**Published:** 2018-04-10

**Authors:** Dennis O. Ndolo, Michael Wach, Patrick Rüdelsheim, Wendy Craig

**Affiliations:** ^1^International Centre for Genetic Engineering and Biotechnology, Cape Town, South Africa; ^2^Michael Wach Consulting, Salem, OR, United States; ^3^Perseus BVBA, Sint-Martens-Latem, Belgium; ^4^International Centre for Genetic Engineering and Biotechnology, Trieste, Italy

**Keywords:** biosafety, eLearning, risk analysis, distance education, curriculum, biorisk management, food safety, environmental safety

## Abstract

Anyone working in biosafety capacity enhancement faces the challenge of ensuring that the impact of a capacity enhancing activity continues and becomes sustainable beyond the depletion of funding. Many training efforts face the limitation of one-off events: they only reach those people present at the time. It becomes incumbent upon the trainees to pass on the training to colleagues as best they can, whilst the demand for the training never appears to diminish. However, beyond the initial effort to establish the basic content, repeating capacity enhancement events in different locations is usually not economically feasible. Also, the lack of infrastructure and other resources needed to support a robust training programme hinder operationalizing a “train-the-trainer” approach to biosafety training. One way to address these challenges is through the use of eLearning modules that can be delivered online, globally, continuously, at low cost, and on an as-needed basis to multiple audiences. Once the modules are developed and peer-reviewed, they can be maintained on a remote server and made available to various audiences through a password-protected portal that delivers the programme content, administers preliminary and final exams, and provides the administrative infrastructure to register users and track their progress through the modules. Crucial to the implementation of such an eLearning programme is an approach in which the modules are intentionally developed together as a cohesive curriculum. Once developed, such a curriculum can be released as a stand-alone programme for the training of governmental risk assessors and regulators or used as accredited components in post-graduate degree programmes in biosafety, at minimal cost to the government or university. Examples from the portfolio of eLearning modules developed by the International Centre for Genetic Engineering and Biotechnology (ICGEB) are provided to demonstrate these key features.

## Introduction

Modern biotechnology refers to a number of techniques that involve the intentional manipulation of genes in a predictable and controlled manner, but beyond normal breeding barriers, to generate changes in the genetic make-up of an organism. Such techniques offer great potential for meeting critical needs for food, agriculture, health, and sustainable socio-economic development. However, since modern biotechnology may result in the production of novel organisms, many regulatory authorities worldwide regulate these products as potential biohazards, in an effort to ensure human and environmental safety. Consequently, risk assessments are required before any activity involving them is performed, and only when safety has been duly demonstrated, can they be made commercially available. There are also a number of other organisms that can be exploited by humans for a range of activities; some of which, if not properly handled, could cause harm, either directly, or indirectly, resulting in considerable health, environmental, social, and economic losses. In addition, there are increased threats from the spread of weeds, pests, and pathogens, due to the rapid surge in global movement of people, goods, and organisms coupled with the growing security interest in the potential of organisms as agents for bioterrorism. In light of these concerns, national legislation, as well as various international agreements such as the International Health Regulations, 2005; United Nations Security Council Resolution 1540; the Biological and Toxin Weapons Convention, 1975 (Sture et al., 2013); the International Food Standards of the Codex Alimentarius Commission 1999 (FAO, 1999); and the Cartagena Protocol on Biosafety to the Convention on Biological Diversity, 2000 (CBD, 2000), require that measures—including the provision of relevant education and training—are put in place to prevent harm from biological material. As a result, there is global recognition for the need to develop international biosafety and biosecurity capacity, spanning many sectors, and disciplines, and especially in the developing world and for those countries with economies in transition. Biosafety and biosecurity are related concepts, in that both focus on measures to ensure protection from adverse effects associated with biological material. While biosafety pertains to the protection of human health and the environment from the possible adverse effects of the products of modern biotechnology (CBD, 2000) and is generally used to describe frameworks encompassing the policy, regulation, and management to control potential risks associated with the use of the technology (FAO, 2006), the term biosecurity is most commonly used to refer to mechanisms to establish and maintain the security and oversight of pathogenic microorganisms and toxins to prevent possible misuse; with due attention to all relevant information, knowledge, processes, practices, and equipment associated with potentially or actually hazardous biological material (Sture et al., 2013). An integrated biosafety and biosecurity training curriculum would therefore enable countries to meet their obligations under the above international agreements, and at the same time build their national capabilities to effectively address their own biorisk threats.

Over the years, the Biosafety Group of the International Centre for Genetic Engineering and Biotechnology (ICGEB) has been addressing this need through the development and implementation of a comprehensive educational and training programme in biosafety. This programme has led to the development of highly-skilled and trained personnel whom regulatory authorities can rely upon to ensure there is full and balanced consideration of biosafety issues in pursuing appropriate uses of modern biotechnology. The training programme has involved *inter alia*, the development and establishment of post-graduate programmes at both the Universities of Aberystwyth (UK) and Adelaide (Australia), crucially with essential biosafety components, together with the provision of financial support to a number of African regulatory officials to undertake the programmes. In addition, the Biosafety Group has organized numerous workshops around the world providing basic and advanced training and mentorship to further develop biosafety regulatory capacity.

However, the impact of these face-to-face approaches may be difficult to sustain once funding has been depleted, and repeating such training events in different locations is both difficult and costly. As a result, the Biosafety Group explored alternative learning environments that would provide flexibility and remain available for a prolonged period. One option that was explored was to provide training sessions as webinars, e.g., the American Biological Safety Association regularly organizes webinars (https://absa.org/online-education/) on selected biosafety topics. The advantages offered by this approach is that, compared with face-to-face trainings, a webinar provides broader accessibility, since students and trainers are not required to travel. Also, costs associated with logistics are substantially reduced once the webinar platform has been set up. Nevertheless, this approach still limits the interaction to the single occasion when students and trainers meet virtually.

Another distance learning approach that was investigated involves the use of online discussion fora. For example, the international eLearning postgraduate course “Biosafety in Plant Biotechnology” offered by IPBO and Ghent University (https://studiekiezer.ugent.be/postgraduate-studies-in-biosafety-in-plant-biotechnology-en) combines on-campus training with complementary distance learning in the form of assignments and participation in online discussion groups/chat sessions between students and trainer. This latter aspect allows individual follow-up and in-depth elaboration of specific topics however, although it has less time constraints and is less dependent on punctual access to the Internet, it does limit the active involvement of both the trainers and students to a fixed period.

Other online courses include the Biosafety Practitioner Course (http://www.bti.ed.ac.uk/courses/) and the Professional Course in Biorisk Management (http://www.bti.ed.ac.uk/biorisk-management/), both provided by the Biosafety Training Institute of the University of Edinburgh, UK. These courses are however only offered during specific time periods which limits access. The Centre for Biosecurity of the Public Health Agency of Canada and the Office of Biohazard Containment and Safety of the Canadian Food Inspection Agency offers an eLearning course on Principles of Laboratory Biosafety (https://training-formation.phac-aspc.gc.ca/course/index.php?categoryid=2) which focusses on biosafety in laboratory and containment facilities.

While considering these options, the Biosafety Group began to expand the reach and sustainability of its training programmes, by developing an online eLearning platform for biosafety training. The term eLearning is used here to describe a broad spectrum of internet-based education. By choosing to locate the ICGEB eLearning platform on the cloud (https://showcase-icgeb.elearning.it), it allows users to access content from anywhere with a network connection. It also means that the updating of local IT hardware and software no longer presents technological and financial hurdles, whilst also liberating local providers from bandwidth limitations—a frequent constraint in developing countries. Such a platform therefore ensures that ICGEB biosafety training can be delivered online, globally, at low cost, and on an as-needed basis to multiple audiences. The modules are maintained on a remote server and made available through a password-protected portal that delivers the module content, administers exams, and provides the administrative infrastructure to register users and track their progress. The modules in the ICGEB eLearning platform are being used as stand-alone courses for the training of risk assessors, and as components in post-graduate degree programmes in biosafety, at minimal cost to the hosting government or university.

## Developing a comprehensive and cohesive biosafety curriculum

In order to ensure that all of the key biosafety concepts and training elements are covered, it is necessary that such a training programme is developed as part of a broad-based, cohesive, and comprehensive core curriculum, encompassing all elements common to biosafety and biosecurity regulation. In the development of the eLearning platform, ICGEB has therefore worked closely with established biosafety regulatory offices, institutions, and individuals with strong credentials in biosafety research, education and training, policy, and regulation. Regulatory offices have been involved in the development of the programme to ensure that it has relevance to the needs of GMO regulatory bodies. This element gained increasing importance in preliminary meetings with potential beneficiaries, principally African regulators, who reported that previous efforts to develop such a programme came to naught as they were not tailored to end-user needs. Therefore, from these initial consultations, the following topics were prioritized for development into core eLearning modules:

- **Biosecurity and biosafety—**With the focus on identified biological hazards requiring obvious containment, this introductory module was developed to provide context-setting information, in order to understand the nature of hazards and uncertainties associated with biological material (comprising both GMOs and non-GMOs [principally]). Biosafety and biosecurity are presented as complimentary components of biorisk management, with an especial focus on established practices specific to biosecurity issues.
- **Biosafety regulatory frameworks—**This module introduces users to the different components of a regulatory framework aimed at safeguarding human health and the environment when working with biological material and/or biotechnology applications. A functional framework comprises: legal documents (such as policy, legislation, guidelines, and decisions), authorities, advisory bodies, and enforcement mechanisms. The module guides the user through such a framework, and highlights the importance that it should reflect government policy, which should be coherent with societal priorities and values.
- **Risk analysis—**The purpose of the risk analysis module is to impart knowledge and skills to users to conduct evidence-based risk analysis. At the end of the module, users are able to assess risk using science-based approaches, develop appropriate management options for identified risks, and effectively communicate the risk and appropriate management options to the public and relevant authorities.
- **Containment and confinement of organisms—**Containment and confinement measures can be used when a risk assessment identifies risks that must be managed (e.g., when handling pathogens) or when there is uncertainty on safety (e.g., when developing certain products of biotechnology). This module guides users through the selection, implementation, and verification of various containment and confinement approaches.
- **Environmental safety—**New biological elements can have various impacts in ecosystems. It is necessary that such potential impacts are determined and addressed prior to introducing derived products into the ecosystem. This module focuses on the identification of the most common sources of potential environmental harm from novel biological organisms, as well as the types of analyses that need to be conducted in order to assess the potential risks of such organisms.
- **Food safety—**The objective of this module is to enable users to understand how the safety assessment of food is undertaken in relation to various food-related risks. Internationally-agreed procedures for food safety assessment, as published by Codex Alimentarius, are described in this module and their application discussed. Scientific and public policy issues of relevance to food safety including labeling, traceability and identity preservation of food commodities are also covered.
- **Socio-economic considerations in biosafety decision-making—**Although not a mandatory requirement in decision-making processes in many countries, there is increasing international discussion on the need to consider a broader range of issues, beyond those concerning the environment and health, when assessing the use of novel technologies. This module addresses the possible socio-economic implications of technology adoption, including how these should be addressed within a decision-making context.

As we hope is apparent from the preceding module descriptions, each has been developed to cover key elements and approaches not only specific to biosafety and the regulation of GMOs, but extended to also cover complementary regulatory areas of technology and science (especially, biological). In this way, the portfolio of modules is supportive of the harmonization of similar regulatory approaches, when applicable, as well as being of broader utility to post-graduates seeking employment across government regulatory agencies. The curriculum will be continuously revised as new issues and information emerge so that it stays relevant to emerging biosafety and biosecurity concerns and approaches.

## Design elements of the ICGEB elearning platform

In the design of the eLearning platform, ICGEB needed to address the fact that even high-quality educational content can be undermined by a poorly designed platform. In addition, with today's tech- and media-savvy users, it is not sufficient to simply upload recordings of lecturers giving PowerPoint presentations (e.g., podcasts) onto a website in order to create a meaningful eLearning experience. Considerable thought and effort was invested in the design of the platform itself, to ensure that it not only enhanced the content, but also provided a variety of learning and testing modalities, and was also easy and cost-efficient to administer.

### Design elements to enhance the learning experience

Several features were customized for the eLearning platform to provide a sophisticated and robust interactive learning experience. These focused on:

➢ *Use of multimedia:* Using a variety of media (multimedia) in a learning experience stimulates and encourages users to think more reflectively and helps in maintaining their interest in the learning material (Permanasari et al., 2016). As with many complex scientific topics, the concepts of biosafety are therefore best conveyed to users by exploiting the array of multimedia tools available, and as such, elements were therefore selected within the eLearning platform to accommodate a wide range of media formats. Users have a range of learning styles, and merely displaying text on the screen is almost never a good approach to teaching or to fostering user engagement (Truong, 2016), so the eLearning platform was designed to incorporate static and animated graphic elements, video lectures, interactive diagrams, and external reading material and other resources. The module developer is then free to incorporate these various elements, as needed, to best present the material to the users. For example, a video lecture describing the key features of a Biosafety Level 3 laboratory can be complemented with an interactive diagram of such a lab, in order to reinforce the key points from the presentation and help the users to understand that the features of a BL-3 lab are specifically designed to work together as a system in order to ensure containment.
➢ *Compatibility with mobile technology:* The specific eLearning platform was selected in the acknowledgement that the primary access by users may very well be through the use of mobile technology, e.g., tablets or even smartphones (Viberg and Grönlund, 2017). The platform itself, as well as the presentation of the content, had to support access by a variety of devices, so that each user would always have a high-quality experience, regardless of the device used. This required careful consideration of issues such as font sizes, resolution of graphics, and phrase and paragraph length, so that content would be readable regardless of screen size. For example, quiz questions had to be formatted so that the user would always see the question and all the possible responses without having to scroll down or across the screen.
➢ *Interactive glossary:* Biosafety is fraught with technical terminology, including words such as “confinement,” which have a plain-English definition that is subtly different from its more technological definition when used in a biosafety context. The eLearning platform was therefore designed to incorporate a readily-accessible drop-down glossary into each module, which prompts the user when a new vocabulary item is encountered for the first time, and then would serve as a compendium of all vocabulary items associated across the portfolio, to which the user can refer at any time.
➢ *Exercises and intermediate tests:* Biosafety concepts are challenging to teach and to learn, primarily because many of the high-level concepts are grounded in one or more fundamental concepts. Any misunderstanding of these basic concepts, such as the meaning of *hazard*, may make it impossible for a user to then understand related concepts, such as *risk assessment* and *risk management*, which incorporate and rely on a clear understanding of *hazard*. The eLearning platform was therefore designed to enable the portfolio administrator to assess the degree of user understanding throughout the module, with the aid of short exercises and intermediate tests, as this helps to verify that the user understands all the fundamental concepts before then advancing further into the module with presentations of more complex concepts. Exercises and quizzes can also provide helpful feedback to the user, and even direct the user to review particular sections of the module in order to clarify and resolve any misunderstanding before delving further into the teaching.
➢ *Final exam:* To effectively verify that each user has mastered the key concepts of the module, the eLearning platform enables the content developer and/or host institution to include a final exam, which the user must complete and pass. Currently, the final exam (and any intermediate tests) of each module comprises a number of several types of questions randomly extracted from a much larger large database of questions. These question formats include: true/false, multiple-choice with only one correct answer, multiple-choice with more than one correct answer, ranking questions, and questions using an interactive diagram; and more question formats are under development. Of note, the options from which the user must select the correct answer(s) are randomly displayed each time the question is extracted from the main database. For example, an answer appearing as option A when first extracted, will randomly appear as option A, B, C, or D upon next extraction. In this way, users even when sitting alongside one another and undertaking the exam at the same time, are not presented with the same question set, nor with the answers presented in the same order. For intermediate test only, once the user has completed the questions and received a final grade, the platform enables the user to go back through questions that were not answered correctly, to help them identify topics to review.

The platform can offer learning environments with differing pass rates (of the modules themselves, as well as for the portfolio overall), as well as differing options for re-sitting examinations, tailored to each hosting institution's requirements. To elaborate, each individual hosting institution can dictate: the number of questions from the database to be included in each test and exam, the pass rate for each, the pre-determined waiting period before any exam re-sit, and also the number of times the exams can be re-sat.

Together, these design features allow the creation of sophisticated and rigorous modules, comprising a variety of appropriate media to enhance learning. Previous research has identified a lack of compulsion to engage with online learning material as a potential obstacle to eLearning experiences, given the lack of a formal framework or timetable to which users are accountable (Reid et al., 2016). The ICGEB eLearning platform minimizes the likelihood of such a passive user experience, by incorporating exercises, quizzes, tests, and exam questions to check learning, provide feedback, and increase user engagement.

### Design features to enhance course administration

First and foremost, the eLearning platform was designed to be easy for institutions to adopt. Because the platform is hosted remotely, there are no direct hosting costs or IT staff needs for the institution. Access to the platform, both for users and administrators, is provided through password-protected accounts. The administrator's account provides several functions to facilitate course administration: the authorisation of each user to enroll in the course; the oversight of each user's progress of the course, including user success in preliminary tests and the final exam; the ability to assign modules to users in a pre-determined order, and; the ability to impose deadlines by which all of the users will have completed each module.

Users can quickly and easily track their progress through the course using a personalized “dashboard,” which highlights the overall percentage completion (macro-level) of each assigned module as a circular graph, for example a pie chart or gauge. This is the same when monitoring their progress at the micro-level, i.e., for each chapter in the module, as each of these also has its own gauge to demonstrate the percentage completion. In addition, the dashboard provides user access to external resources and required readings, along with additional resources devised as supplementary module content, and the course compendium glossary.

## Developing elearning materials based on the curriculum

Once the context of the eLearning, i.e., curriculum structure with key learning goals and the design features of the platform were established, a process was put in place to deliver the actual content. In order to allow maximum flexibility, each module was planned to function both on its own and as a part of the larger course curriculum. For each module, at least two experts, internationally-recognized in providing biosafety training, were selected as primary content developers. The first phase consisted of exploratory briefings and exchanges amongst the developers (both content and IT) on the organization and content of the module, and an initial “storyboard” drafted to reflect all of the major elements to be included—this storyboard was continually being updated as the specific content was elaborated and organized. Modules were further divided into chapters and smaller units suitable for eLearning sessions, and especially to facilitate online streaming in narrow-bandwidth geographic areas. Working with smaller units also assisted in interspersing the module content with videos, reading materials, exercises, and other components that allow a user to work at their own pace.

The first phase provided a first level of critical review of content. It would later also result in a diversity of experts presenting the content, making it more engaging to the user. Although the experts had highly-recognized experience on the specific topics and therefore were more than capable of making in-depth presentations, the adaptation of the materials for the purposes of eLearning was an ever-present challenge. Although a general rule when presenting PowerPoint slides is to not use overly-long sentences, the number of words to eventually be displayed on the screen in the eLearning context had to be reduced even more. For complex scientific concepts and legal texts in particular, this was a difficult task, as quotations have to be complete and correct. In such instances, experts were advised to use, as much as possible, complimentary documents, rather than trying to force all of the information into a recorded video. This is one of the aspects where a good exchange amongst the content and IT developers is essential. While the IT developers need to understand the overall objectives of the course, experts need to understand: the capabilities of the platform tools and IT developers (especially in the field of graphics and animation); the limitations of a computer-based presentation, and; a “common language” with the IT developers, so that ambiguities are minimized and everyone understands how the overall objectives are to be achieved collectively.

Initial PowerPoint presentations prepared by the experts were then transferred to, and revised in, eLearning-ready templates. The resulting eLearning-ready presentations included all of the necessary technical indications such as animation, timings, and suggestions for specific graphics. Whenever an external third-party source was required (document, video, picture, etc.), appropriate authorisation was obtained, even if the source was freely-available on the Internet. The eLearning-ready presentations were verified once more by the experts who had developed the materials, and acted as a second level of quality check. This check was also important in order to ensure consistency between the different modules. For example, concepts such as *biosafety* or *biorisk* may have slightly different meanings in different contexts and, if not specifically mentioned, may confuse the user.

Video content was recorded in a professional study, by a cameraman and sound technician. One key observation made by the experts was that recording a video is a very different experience to providing a lecture for a live audience. In spite of the experience of all experts with live audiences, the professional insights of the recording crew were required to allow smooth transitions between slides. With the scenario clearly spelled out in the eLearning storyboard, including the placement of exercises and preliminary tests, the recordings were then tailored to facilitate post-editing. Also, the crew ensured that the atmosphere allowed a natural and relaxed recording, in spite of the many hours confinement to a specific fixed spot under hot lights. At the end of the recording, all results were reviewed with the IT developers and final arrangements were cross-checked for additional materials, glossary, exercises, etc.

The remaining steps were the production of a module “Alpha” version, which was a compendium of all of the integrated recorded and non-recorded information, that was subjected to a third verification and edits by the content and IT developers, before the resulting “Beta” version was subjected to peer-review by external biosafety and biosecurity experts in order to help identify any inaccuracies, contradictions, omissions, or inconsistencies in the content, along with any software faults, bugs or difficulties in access/use. Their comments were addressed by the content and IT developers, eventually leading to the final “production” version of the module.

## Discussions and conclusions

From the perspective of content developers, key take-home messages from the eLearning experience include:

- Learning objectives should be clearly elaborated at the outset to ensure that each module covers all of the intended subject material, and that it interconnects within the overall curriculum.
- Experts should be briefed on the possibilities and limitations of the eLearning platform, in order to prepare content that provides the best learning experience.
- Experts need to be mentally prepared for a non-audience presentation, overcoming the absence of direct contact and feedback from a live audience. The timing of the appearance of images and text is very different.
- Experts must be made aware of the diversity of materials that can be used and should select the best suited format, e.g., background documents for longer texts.
- Consistency is essential and tools, such as a common glossary, should be compiled for all modules and shared by all experts.
- The development of a “common language” and good exchanges between the content and IT developers can result in magic! Discussing the options and clarifying the intentions of a slide may result in powerful images that will better attract the attention of the user.

The current portfolio of biosafety modules offers great promise in the development of autonomous and enduring biosafety systems that are relevant, useful, and resilient. The design of this portfolio encompasses not only GMOs, but also matters related to biosecurity, public health, natural resource management, biocontrol, and bioremediation. In fact, the breadth of coverage offered the portfolio was of especial focus, so that it appeals to users with different access points in technology regulation, including the regulated community, the regulators, and wider stakeholders with roles in regulatory decision-making. The most obvious benefits from utilizing eLearning in biosafety training include:

- **Flexible approach**—Provides hosting institutions as well as users with the flexibility of time and place of delivery.
- **Comprehensive coverage**—Enhances the efficacy of knowledge and qualifications via an ease of access to a huge amount of information.
- **Cost-effectiveness**—No need for training recipients (i.e., users) to travel to training venues. Also offers learning opportunities to a maximum number of users, without the need for education premises and facilities.
- **User-friendly**—Takes into consideration user's individual learning styles and paces.
- **Sustainable**—Helps compensate for any local scarcities of experts, allowing hosting institutions to take charge of local training needs, at minimal expense, and staff time investment.

Efforts are underway to not only offer the current portfolio of eLearning modules in courses to additional hosting institutions around the world, but also to extend the portfolio (and thus possible courses) through the development of modules covering complementary topics and approaches, especially in the field of government oversight of technological applications in biology-based sectors. In addition, investigations are being made into the availability of additional IT tools and accessories to help enhance the user educational experience, especially at the local level, and to continue tailoring the offered training to the future needs and potentials of the target user communities and hosting institutions.

## Author contributions

All authors listed have made a substantial, direct and intellectual contribution to the work, and approved it for publication.

### Conflict of interest statement

MW is employed by Michael Wach Consulting, while author PR is employed by Perseus BVBA. All other authors declare that the research was conducted in the absence of any commercial or financial relationships that could be construed as a potential conflict of interest.
